# 

**Published:** 1951-04

**Authors:** 


					PUBLISHED UNDER THE AUSPICES OF THE BRISTOL MEDICO-CHIRURGICAL SOCIETY
THE BRISTOL
MEDICO - CHIRURGICAL
JOURNAL
A Journal of the Medical Sciences for the
WEST OF ENGLAND
AND SOUTH WALES
, 11 ,MI
Editor: ^ j - y
E. WATSON-WILLIAMS, M.C., M DyEw,, ifSfesr
with whom are associate<f/ MEDICAL
G. E. F. SUTTON, M.C., M.0., F.R.C.P.
J. H. MIDDLEMISS, M.D., D.M.R.D., F.F.AL)N
N. S. CRAIG, M.C., M.D., Alston ?Jitor|^ ??-p-
local Correspondents: ^ggk? -
Bath?A. D. BATEMAN, O.B.E., F.R.C.S.
Exeter?L. N. JACKSON, M.C., D.M.
Gloucester?R. F. JARRETT, M.B., M.R.C.P.
Newport?J. L. D. WILLIAMS, M.B., F.R.C.S.
Plymouth?C. R. CROFT, D.M., M.R.C.P. Taunton?Dr. M. McALLEY, D. M.R.
Torquay?Dr. A. ROBINSON THOMAS, D.M.R.E.
Truro?Dr. F. D. M. HOCKING, F.R.I.C. Yeovil?J. A. VERE NICOLL, F.R.C.S.
I
BRISTOL :
J. W. ARROWSMITH LTD., QUAY STREET AND SMALL STREET
1^
I01* LXVm. No. 246. Price: FOUR SHILLINGS net.
APRIL ? 1951

				

